# The demographics of vaccine hesitancy in Shanghai, China

**DOI:** 10.1371/journal.pone.0209117

**Published:** 2018-12-13

**Authors:** Jia Ren, Abram L. Wagner, Anna Zheng, Xiaodong Sun, Matthew L. Boulton, Zhuoying Huang, Brian J. Zikmund-Fisher

**Affiliations:** 1 Department of Immunization Program, Shanghai Municipal Centers for Disease Control & Prevention, Shanghai, China; 2 Department of Epidemiology, School of Public Health, University of Michigan, Ann Arbor, Michigan, United States of America; 3 School of Information, University of Michigan, Ann Arbor, Michigan, United States of America; 4 Department of Internal Medicine, Division of Infectious Disease, University of Michigan Medical School, Ann Arbor, Michigan, United States of America; 5 Department of Health Behavior and Health Education, School of Public Health, University of Michigan, Ann Arbor, Michigan, United States of America; 6 Department of Internal Medicine, Division of General Medicine, University of Michigan Medical School, Ann Arbor, Michigan, United States of America; University of Campania, ITALY

## Abstract

**Background:**

Vaccine hesitancy has been little studied in low- and middle-income countries but is a potential concern because vaccine refusal may increase the burden of infectious diseases and impede control efforts. The aim of this study was to compare vaccine hesitancy between locals, long-time city residents, and non-locals, who have more recently moved to the city from either other urban or rural areas, in Shanghai, China.

**Methods:**

Parents of infants ≤3 months of age were surveyed at immunization clinics in Shanghai, China. Participants completed a paper questionnaire utilizing the 10-item Vaccine Hesitancy Scale, which was developed by the World Health Organization Strategic Advisory Group of Experts on Immunization. Items were grouped based on internal consistency, and regressed onto demographic variables using a negative binomial model.

**Results:**

In total, 1,188 (92.5%) individuals participated. For most items on the scale, parents expressed positive beliefs about vaccines. However, about half of parents somewhat or strongly agreed that new vaccines carried more risks than older vaccines, and 71.6% somewhat or strongly agreed that they were concerned about serious adverse effects. Seven items from the Vaccine Hesitancy Scale were highly correlated and mapped onto “lack of confidence”; the other three items were analysed separately. Compared to mothers, fathers had less lack of confidence (β: -0.06, 95% CI: -0.12, -0.01), and individuals living in the outer suburbs (β: 0.13, 95% CI: 0.01, 0.25) and rural non-locals (β: 0.10, 95% CI: 0.02, 0.18) had greater lack of confidence in vaccines compared to their urban or local counterparts, respectively.

**Discussion:**

Shanghai parents professed confidence in certain vaccine benefits, but vaccine messaging could focus on addressing misconceptions about vaccines for diseases no longer common, newer vaccines, and adverse effects associated with vaccination. These messages may need to be separately tailored to locals and non-locals, who have differing concerns.

## Introduction

The provision of vaccines through the government-funded Expanded Program on Immunization (EPI) has been a successful means of controlling many infectious diseases globally [1,2]. China’s EPI was started in 1978 and initially included vaccines for tuberculosis, polio, measles, pertussis, diphtheria, and tetanus, and was later expanded to include several additional vaccines in 2002 and again in 2007 [1].

Progress in the control of vaccine-preventable diseases can by stymied by vaccine hesitancy and resultant low uptake of vaccines. A World Health Organization (WHO) Strategic Advisory Group of Experts on Immunization (SAGE) working group defines vaccine hesitancy as a “delay in acceptance or refusal of vaccines despite availability of vaccination services. Vaccine hesitancy is complex and context specific varying across time, place and vaccines. It includes factors such as complacency, convenience and confidence” [3].

The relationship between individual decisions not to vaccinate and population-level outbreaks is particularly relevant for highly infectious diseases like measles and pertussis [4,5]. Vaccine hesitancy has been predominantly studied in high income countries [6,7], although some limited information from low- and middle-income countries (LMICs) is available [8]. One review found that, in 2016, national assessments of vaccine hesitancy were undertaken in 47% of low income countries, 45% of lower middle income countries, 22% of upper middle income countries, and 53% of high income countries [9]. Clearly, more information is needed from upper middle income countries like China.

Vaccine hesitancy is likely to vary by demographic conditions, educational background, and experiences with diseases and vaccines. Residency is one such grouping: non-locals, sometimes referred to as the “floating population,” are migrants from rural areas to urban cities (“rural non-locals”) or from other urban areas in China to another city (“urban non-local”) [10], and typically have less access to governmental entitlement programs than locals [10,11]. However, non-local children, like locals, receive all EPI vaccines for free. Of China’s 1.3 billion residents, 260 million are non-locals; and in Shanghai, 9 of 23 million residents are non-locals. Non-locals often cluster within neighborhoods in suburban districts of cities because of affordability and location of jobs: urban districts represent historical business areas, whereas suburban districts are more industrial. Regardless of residency, health statuses in suburban areas are expected to be poorer than urban areas because of lower individual socioeconomic status and because there is less access to public services in these areas [12,13]. Because of their socioeconomic and legal circumstances, it is highly plausible that medical decision-making, including decisions about vaccines, differs between locals and non-locals [14].

More information is needed about the development of vaccine hesitancy in LMICs like China, and within special populations, like non-locals or migrants. The aim of this study was to describe the features of vaccine hesitancy among parents of young infants and to compare vaccine hesitancy across different demographic groups, in particular between locals and non-locals in Shanghai, China.

## Methods

### Study population

This cross-sectional study utilized a stratified, two-stage cluster sample in Shanghai, China. The first stage was at the township level with city districts forming a sampling strata, such that at least one township was selected from 15 districts (i.e., all districts in the municipality with the exception of Chongming district, which is an insular, less densely-populated district). We selected 40 townships based on the population of children in the township according to the 2010 China Census. Within each township, study staff visited an immunization clinic and selected 30 caregivers (preferentially mothers or fathers) based on a convenience sample of who was visiting the clinic that day. Every township has an immunization clinic, and, therefore, the catchment area for the immunization clinic is coterminous with the township. The inclusion criterion was a parent or grandparent of a child <3 months of age. All participants had to be at least 18 years.

The sample size calculation was estimated based on another aim of the study: to evaluate longitudinal relationships between vaccine hesitancy and vaccine uptake. Based on a previous survey looking at uptake of pneumococcal vaccination—a non-EPI vaccine which requires payment (data unpublished), we found that those who thought that the vaccine was more necessary had an uptake of 13.90%, whereas those who perceived the vaccine as unnecessary had an uptake of 5.49%. A sample size of 191 in each group (i.e. those who think necessary and those who think unnecessary) would be required to see a statistically significant relationship of this size given 80% power and a 95% confidence level. Assuming 50% in each category, this would mean we would need 382 individuals. Given an intra-cluster coefficient of 0.02389 (estimated from this previous data source), that meant 647 individuals needed to be enrolled.

Data collection occurred between May and September 2017.

### Vaccine Hesitancy Scale

**Fig 1:**
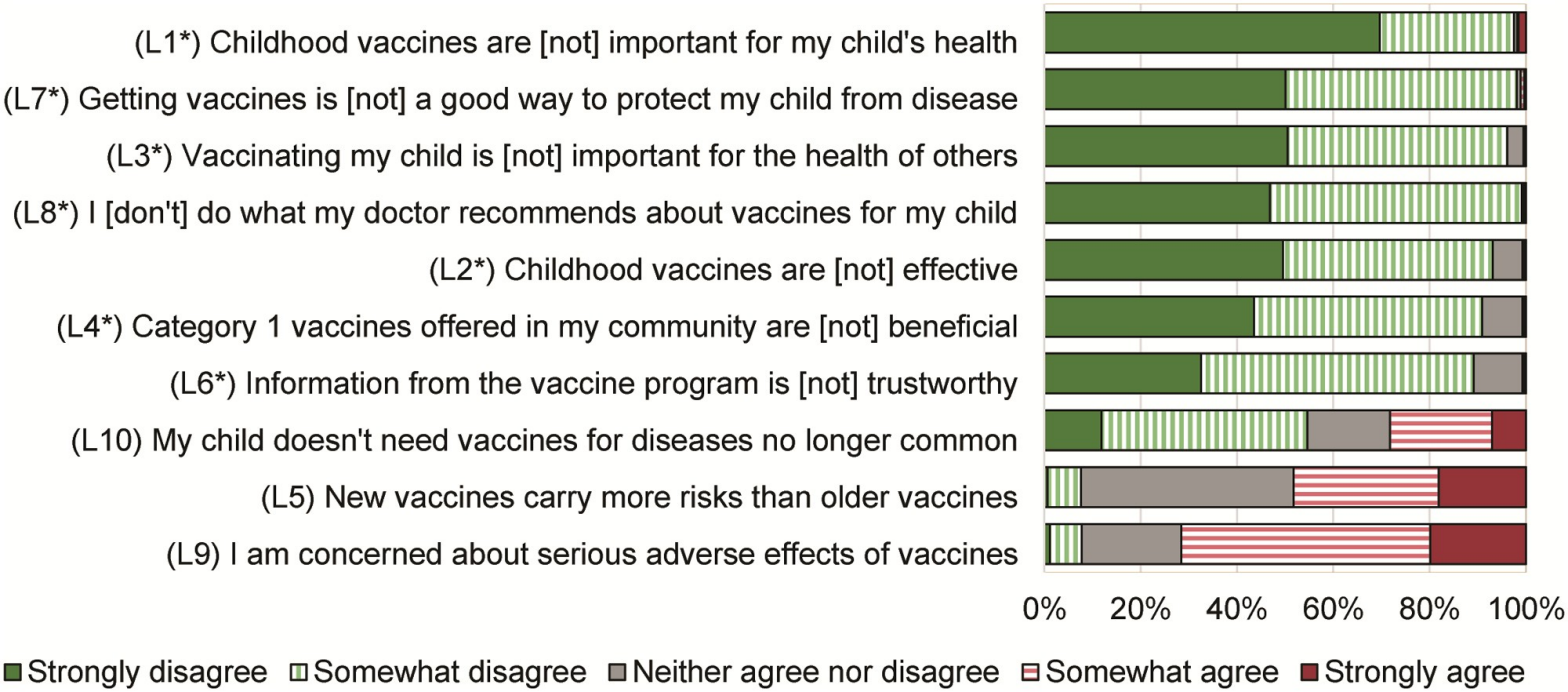
Distribution of responses to each item of the vaccine Hesitancy Scale, Shanghai, 2017.

The 10-item Vaccine Hesitancy Scale was developed by the WHO SAGE Working Group to address Vaccine Hesitancy [15]. Each item was assessed on a 5-point Likert scale; to make directionality uniform across all items, some items’ response values were flipped, such that the highest number represented more hesitant beliefs. Based on internal consistency of scale items and from considerations of previous research [16], seven items (L1-L4, L6-L8) were grouped into the component “Lack of confidence,” and the other three items were analyzed separately. To create a scale for the component “lack of confidence,” the average of all seven items was taken in order to preserve the original 1–5 scale. The other three items (concerns about side effects, newer vaccines, and the continued use of vaccines for relatively controlled diseases) were analyzed individually. Fig 1 contains an English translation of all questions.

### Statistical analysis

The distribution of demographic characteristics is displayed using descriptive statistics. We assessed the differences in the proportion of various demographic characteristics between locals and non-locals through a Rao-Scott chi-square test. Internal consistency of the scale was analyzed using Crohnbach’s alpha. We regressed components of the Vaccine Hesitancy Scale onto demographic characteristics using generalized estimating equations with a negative binomial distribution and identity link and using an exchangeable correlation matrix. This regression model output β estimates that represent the increase in hesitancy for a given change in the dependent variable.

Analyses used weighting and survey procedures when possible. The sampling weights derived from the complex sampling scheme and the inverse probability of selection for each participant. The weights are designed to make the study population generalizable to the population of parents of young infants in Shanghai. The analyses also clustered observations by township. We assessed significance of results an alpha level of 0.05, and precision of results through 95% confidence intervals (CI).

### Ethical approval

The study protocol was approved by the University of Michigan Health Sciences and Behavioral Sciences Institutional Review Board (#HUM00125379) and an ethical review committee from the Shanghai Centers for Disease Control and Prevention (#2017–2). Participants gave written informed consistent prior to data collection.

## Results

In total, 1,285 individuals were approached to participate and 1,188 (92.5%) consented and were enrolled in the study. Table 1 shows the demographic distribution of the study population. Locals were 48.3% of the population; non-locals formed the remainder, with 15.8% originally from urban areas and 35.9% originally from rural areas., Some characteristics varied by residency status with urban and rural non-locals comprising a larger proportion of the population in the inner and outer suburbs than in the urban districts (P<0.0001). Additionally, rural non-locals reported less personal experience with vaccine-preventable diseases (22.8%) compared to locals (24.8%) and urban non-locals (32.9%) who had similar proportions (P = 0.0021).

**Table 1:** Demographic differences between locals and non-locals in Shanghai, 2017.

		Local	Urban non-local	Rural non-local	
	Count (%)	Count (%)	Count (%)	Count (%)	P^a^
**Overall**	1,188 (100%)	552 (100%)	216 (100%)	411 (100%)	
**Relation to child**					0.5569
**Mother**	784 (67.5%)	370 (67.1%)	143 (64.7%)	271 (69.2%)	
**Father**	355 (31.6%)	159 (31.5%)	67 (34.5%)	129 (30.6%)	
**Grandparent**	12 (0.9%)	9 (1.4%)	2 (0.8%)	1 (0.3%)	
**Sex of child**					0.0975
**Girl**	541 (46.6%)	262 (49.5%)	95 (44.1%)	184 (43.9%)	
**Boy**	601 (53.4%)	270 (50.5%)	114 (55.9%)	217 (56.1%)	
**Urbanicity**					<0.0001
**Urban**	357 (31.1%)	230 (43.6%)	54 (26.0%)	73 (16.6%)	
**Inner suburb**	596 (40.3%)	232 (29.2%)	130 (54.2%)	234 (49.0%)	
**Outer suburb**	226 (28.6%)	90 (27.3%)	32 (19.8%)	104 (34.3%)	
**Education**					<0.0001
**≤Middle school**	198 (21.3%)	23 (7.6%)	17 (8.6%)	158 (45.5%)	
**High school**	168 (14.5%)	25 (4.5%)	23 (10.8%)	120 (29.7%)	
**Vocational**	257 (24.0%)	126 (29.1%)	51 (24.1%)	80 (17.1%)	
**College**	437 (31.4%)	290 (44.4%)	105 (48.7%)	42 (6.0%)	
**Graduate school**	113 (8.8%)	87 (14.4%)	20 (7.7%)	6 (1.7%)	
**Monthly family income**					<0.0001
**<5,000 RMB**	168 (17.7%)	47 (11.5%)	18 (8.7%)	103 (30.2%)	
**5,000–7,499 RMB**	226 (20.6%)	60 (12.4%)	33 (17.3%)	133 (33.2%)	
**7,500–9,999 RMB**	172 (16.0%)	86 (17.7%)	24 (11.7%)	62 (15.7%)	
**10,000–14,999 RMB**	238 (20.4%)	138 (25.0%)	52 (24.2%)	48 (12.5%)	
**≥15,000 RMB**	349 (25.2%)	212 (33.4%)	84 (38.1%)	53 (8.3%)	
**Experience with VPDs**					0.0021
**No**	790 (69.8%)	346 (65.2%)	138 (67.1%)	306 (77.2%)	
**Yes**	389 (30.2%)	206 (34.8%)	78 (32.9%)	105 (22.8%)	

VPD, vaccine-preventable diseases

^a^ From Rao-Scott chi-square test of the difference in proportions among locals, urban non-locals, and rural non-locals.

Fig 1 shows the distribution of responses for each item on the scale. A large majority of parents (69.7%) strongly agreed that vaccines are important for their child’s health (item L1), and about half strongly agreed that vaccines are a good way to protect their child from disease (item L7) and that vaccinating their child is important for the health of others (item L3). For most other items, parents expressed positive beliefs about vaccines. However, about half of parents somewhat or strongly agreed that new vaccines carried more risks than older vaccines (item L5), and 71.6% somewhat or strongly agreed that they were concerned about serious adverse effects (item L9).

Internal consistency was acceptable for the entire 10-item scale (Crohnbach’s alpha = 0.73). It was improved by removing items L5, L9, and L10. The other 7 items had good internal consistency (Crohnbach’s alpha = 0.89) and mapped onto the component “lack of confidence.” The other three items had poor internal consistency (Crohnbach’s alpha = 0.48 for L5, L9, and L10, or 0.37 for L5 and L9 without L10). Accordingly, these three other items were analyzed separately. Details of the factor loadings from an exploratory and confirmatory factor analysis are in S1 File.

Multivariable regression model results for the “lack of confidence” component and three other items are shown in Table 2. For each model, positive β estimates represent greater degrees of hesitancy and negative β estimates represent less hesitancy, relative to reference group. We observed significant socioeconomic differentials for all four measures of vaccine hesitancy. However, the item “concerns about side effects” had the least amount of variation, while also having the highest intercept, indicating that most individuals expressed a relatively large amount of concern, regardless of their background.

**Table 2:** Characteristics related to four different components of vaccine hesitancy according to negative binomial models, Shanghai, 2017.

	Lack of confidence (items L1-L4, L6-L8)		New vaccines risky (item L5)		Concerns about side effects (item L9)		Some vaccines no longer needed (item L10)	
	β (95% CI)	P	β (95% CI)	P	β (95% CI)	P	β (95% CI)	P
**Intercept**	1.58 (1.41, 1.75)	<0.0001	3.13 (2.95, 3.31)	<0.0001	4.03 (3.71, 4.34)	<0.0001	3.00 (2.74, 3.26)	<0.0001
**Relation to child**								
**Mother**	reference		reference		reference		reference	
**Father**	-0.06 (-0.12, -0.01)	0.0179	0.21 (0.07, 0.36)	0.0030	-0.13 (-0.25, -0.01)	0.0311	0.06 (-0.08, 0.20)	0.3884
**Grandparent**	0.16 (-0.06, 0.39)	0.1588	-0.04 (-0.37, 0.30)	0.8320	-0.34 (-0.84, 0.17)	0.1911	0.12 (-0.43, 0.66)	0.6704
**Urbanicity**								
**Urban**	reference		reference		reference		reference	
**Inner suburb**	0.03 (-0.08, 0.15)	0.5935	0.00 (-0.14, 0.14)	0.9737	-0.01 (-0.17, 0.16)	0.9495	-0.17 (-0.47, 0.14)	0.2809
**Outer suburb**	0.13 (0.01, 0.25)	0.0413	-0.10 (-0.19, -0.01)	0.0296	-0.13 (-0.31, 0.06)	0.1837	-0.30 (-0.61, 0.01)	0.0606
**Residency**								
**Local**	reference		reference		reference		reference	
**Urban non-local**	0.07 (-0.02, 0.16)	0.1138	-0.15 (-0.28, -0.01)	0.0293	-0.06 (-0.32, 0.20)	0.6473	-0.29 (-0.53, -0.05)	0.0174
**Rural non-local**	0.10 (0.02, 0.18)	0.0128	-0.22 (-0.41, -0.04)	0.0189	-0.10 (-0.25, 0.05)	0.1890	-0.34 (-0.52, -0.16)	0.0002
**Education**								
**≤Middle school**	-0.06 (-0.22, 0.10)	0.4474	0.49 (0.21, 0.77)	0.0006	-0.21 (-0.52, 0.11)	0.1986	-0.16 (-0.76, 0.43)	0.5914
**High school**	0.00 (-0.15, 0.15)	0.9776	0.53 (0.22, 0.83)	0.0006	-0.24 (-0.48, 0.00)	0.0527	-0.06 (-0.56, 0.44)	0.8073
**Vocational**	-0.09 (-0.24, 0.06)	0.2294	0.47 (0.26, 0.69)	<0.0001	-0.13 (-0.38, 0.12)	0.3222	-0.12 (-0.48, 0.23)	0.4913
**College**	-0.13 (-0.28, 0.01)	0.0716	0.38 (0.22, 0.54)	<0.0001	-0.09 (-0.40, 0.22)	0.5679	-0.02 (-0.34, 0.31)	0.9058
**Graduate school**	reference		reference		reference		reference	
**Monthly family income**								
**<5,000 RMB**	0.03 (-0.06, 0.12)	0.5125	0.09 (-0.15, 0.32)	0.4672	0.13 (-0.08, 0.34)	0.2192	0.21 (-0.12, 0.54)	0.2109
**5,000–7,499 RMB**	-0.10 (-0.23, 0.03)	0.1168	0.14 (-0.03, 0.32)	0.1125	0.12 (-0.07, 0.32)	0.2187	0.05 (-0.38, 0.48)	0.8204
**7,500–9,999 RMB**	-0.02 (-0.12, 0.08)	0.7128	0.33 (0.14, 0.51)	0.0007	0.15 (-0.10, 0.39)	0.2385	0.22 (-0.14, 0.58)	0.2267
**10,000–14,999 RMB**	0.08 (-0.02, 0.17)	0.1275	0.07 (-0.11, 0.25)	0.4591	0.09 (-0.11, 0.29)	0.3640	0.11 (-0.13, 0.35)	0.3842
**≥15,000 RMB**	reference		reference		reference		reference	
**Experience with VPDs**								
**No**	reference		reference		reference		reference	
**Yes**	0.00 (-0.08, 0.08)	0.9304	0.00 (-0.13, 0.13)	0.9932	-0.07 (-0.21, 0.06)	0.3035	-0.12 (-0.26, 0.02)	0.0948

Residency was a significant predictor for all vaccine hesitancy measures, except for “concerns about side effects.” There was no significant difference in the lack of confidence between locals and urban non-locals, but rural non-locals had less confidence than locals (β: 0.10, 95% CI: 0.02, 0.18). However, compared to locals, both urban and rural non-locals had less concerns about new vaccines being risk or about some vaccines no longer being needed.

There were also significant differences by parent type, education, and income. Compared to mothers, fathers had more confidence (β: -0.06, 95% CI: -0.12, -0.01) and were less concerned about side effects (β: -0.13, 95% CI: -0.25, -0.01). However, they also expressed greater beliefs that new vaccines were riskier than older ones (β: 0.21, 95% CI: 0.07, 0.36). Education and income was related to beliefs about new vaccines being risky, but not the other vaccine hesitancy measures. Individuals with lower levels of education or lower income levels had greater degrees of belief that new vaccines were riskier. For example, compared to those with a graduate school education, those with a high school education were 0.53 points higher on the 5-point Likert scale (95% CI: 0.22, 0.83).

## Discussion

Vaccine hesitancy is a globalized phenomenon with the potential consequence of impeding efforts to control or eradicate preventable but potentially debilitating diseases. This study, based in Shanghai, a populous area undergoing rapid economic development and is, consequently, a destination for many internal migrants, found that parents expressed positive beliefs about certain aspects of vaccination. However, those positive perceptions were tempered among most parents by concerns about adverse events of vaccination and doubts about both newer vaccines and vaccines for diseases that were no longer common.

We found that residency was a strong predictor of vaccination beliefs. Individuals with a rural, non-local residency permit had less belief in vaccine benefits but were also less concerned about side effects and vaccines for uncommon diseases. These beliefs were not influenced by experience with vaccine-preventable diseases. Interestingly, the proportion of individuals with personal experience having a vaccine-preventable disease varied significantly by residency, with rural non-locals much less likely to have such experience.

In summary, the group of individuals who had more experience with vaccine-preventable diseases (locals) also had more confidence in vaccines while also expressing more concerns in the riskiness of new vaccines and in the utility of certain vaccines. In a previous study in Shanghai, we also did not observe a relationship between experience with disease and perceptions of vaccine necessity [17]. Although we had initially hypothesized that exposure to vaccine-preventable disease would predispose an individual to wanting that vaccine, other research has shown that the opposite association can exist. Researchers in Arizona, for example, found that parents who obtained a nonmedical vaccine exemption for their child (and who could therefore be labeled as relatively vaccine hesitant) were much more likely to report knowing someone who had a vaccine-preventable disease compared to those who did not obtain an exemption [18].

Our findings had both similarities and differences to a previously published paper analyzing differences across demographic groups using this scale [16]. We have found the opposite association compared to the Canadian study [16], in that our study showed fathers to have more confidence in vaccines but heightened concern about certain vaccine risks compared to mothers. However, similar to the Canadian study, we found that individuals with less education had more concerns about newer vaccines. Although there is rising concern globally that higher educated groups express more concerns about vaccines and more frequently seek exemptions [19], this is not a uniform pattern [18]. In past research, we have also found that some higher educated groups in Shanghai have more concerns about vaccine scheduling [20], but in this study, we did not find many significant differences by education, except that lower educated groups had more concerns about the riskiness of new vaccines. One systematic review of studies from LMICs posits that maternal education directly impacts vaccination awareness and attitudes [21]. And results from our study suggest that vaccination providers may be more effective if they tailor their discussion of vaccines to individuals’ educational levels with special consideration given to discussing the importance of new vaccines and their associated risks with individuals who are less educated.

### Public health significance

The three items that did not map onto the component “lack of confidence” represent important areas for public health officials to promote. Caregivers show clear concerns about side effects, newer vaccines, and the continued use of vaccines for relatively controlled diseases. These concerns are not exclusive to the general population; qualitative interviews with doctors in Alberta, Canada, found that health care professionals, while overall holding very positive beliefs (or attitudes) about vaccines also expressed reservations about the newness of certain vaccines, in addition to distrust of pharmaceutical companies and of vaccine schedules [22]. Therefore, from a societal perspective, limiting the diffusion of vaccine hesitancy will rely on education of vaccination providers on both the benefits of vaccines and on best practices to communicate with parents and other caregivers. Doctors are trusted sources of health information and are therefore pivotal in promoting the use of vaccines [23,24]. For example, in a series of studies from Italy, information from physicians was associated with greater knowledge of human papillomavirus vaccination in pregnant women [25], adolescent and young adult men [26], and parents of young men [27]. However, in order to avoid exacerbating intransigency from caregivers, vaccination providers should be able to briefly provide caregivers with facts about the safety of vaccines, and be willing to ask open-ended questions and listen to the concerns of parents [28].

Caregivers in this study believed vaccines were important for both their own child’s health and for the health of others. It follows that another tactic—to increase confidence in vaccines among caregivers and promote their decision to obtain a vaccine for a child—would be for vaccination providers to talk with them about how some children have a contraindication to vaccination, and so vaccinating one’s own child can have a positive impact on other community members.

### Use of vaccine Hesitancy Scale

Our future research will focus on the relationship between vaccine hesitancy, as quantified through baseline results in this Vaccine Hesitancy Scale, and parents’ decision to obtain mandatory and non-mandatory vaccines. In the absence of this predictive validity, we advise the use of the scale with the following observations: Similar to studies in Canada [16] and Guatemala [29], we found that the Vaccine Hesitancy Scale, as developed by Larson et al. [15], had adequate internal consistency, which was improved by removing items L5, L9, and L10. Unlike the Canadian study, we did not think that items L5 and L9 were correlated highly enough to combine into one construct. We also remark that these three items (L5, L9, and L10) are worded in the opposite direction from other items; that is, a higher value of response in the original survey for these items indicated higher vaccine hesitancy, whereas higher values of response for other items indicated lower vaccine hesitancy. Individuals quickly completing a survey may mistake this directionality. To counter this issue and minimize bias, the order of questions could be randomized, and, if appropriate, the directionality of certain questions could be randomly flipped for some respondents.

### Strengths and limitations

This study has several strengths and limitations. As a convenience sample, we are likely to have a population biased towards accepting vaccines and being more health conscious. However, we note that vaccines are mandatory in China for school entry, and in one study of migrant parents in Beijing, only 11.9% had not gone to an immunization clinic [30]. In addition, although residency status is an objective measure in that it is an official government designation, individuals can change their residency status (for example, through education or an official job). In this way our study could have found a diluted association between residency status and hesitancy if non-locals are transitioning into being locals. A strength of this study is that townships were selected throughout Shanghai, generating a population representative of city. Additionally, by selecting caregivers of young children (<3 months of age), we were able to interview parents at an early point in their child’s life, when they have likely had little prior exposure to vaccinations. A future longitudinal study could examine how attitudes towards vaccinations change in this population as the parents are exposed to more material from doctors, social media, and news media.

## Conclusions

This study used a Vaccine Hesitancy Scale, developed by a the WHO SAGE [15] and validated within a Canadian population [16], to characterize vaccine confidence and beliefs about certain risks within Shanghai, China. China’s ability to add more vaccines into their vaccination schedule and to promote the distribution of non-mandatory vaccines could be limited by concerns about adverse events following immunization and the risks of newer vaccines. Countering these concerns will be particularly important in lower educated groups and among non-locals who have more recently moved into a city. Rapid urbanization is a hallmark of many LMICs, and so contrasting and comparing the values that migrants and non-migrants hold will be increasingly important when developing health promotion programs in China and other countries [31].

## Supporting information

S1 File Factor loadings from the vaccine Hesitancy Scale. (PDF)
