# Supplementary material for: The demographics of vaccine hesitancy in Shanghai, China
Source: PLoS One. 2018 Dec 13;13(12):e0209117. doi: 10.1371/journal.pone.0209117 (PMC6292563; doi:10.1371/journal.pone.0209117)
Supplement: S1 File — (PDF) [file pone.0209117.s001.pdf]

S1 File

Factor loadings from the Vaccine Hesitancy Scale (as developed by the World Health Organization Strategic Advisory Group of Experts on Immunization (https://doi.org/10.1016/j.vaccine.2015.04.037) are displayed below. This sample of caregivers from Shanghai is compared with a sample of Canadian parents, as reported by Shapiro et al. (https://doi.org/10.1016/j.vaccine.2017.12.043).

For the purposes of comparison, we followed Shapiro et al.'s lead in deleting item 10 from the analysis. The study population was divided into two, with one half (n=546) being analyzed with an Exploratory Factor Analysis (EFA) with varimax orthogonal rotation, and the other half (n=538) being analyzed with a Confirmatory Factor Analysis (Table 1).

S1 Table. EFA loadings and CFA standardized loadings.

	Exploratory Factor Analysis				Confirmatory Factor Analysis			
	Shanghai Sample		Canadian Sample		Shanghai Sample		Canadian Sample	
	Factor 1: Lack of confidence	Factor 2: Risks	Factor 1: Lack of confidence	Factor 2: Risks	Factor 1: Lack of confidence	Factor 2: Risks	Factor 1: Lack of confidence	Factor 2: Risks
**L1**	0.671	0.152	0.864	0.195	0.522		0.868	
**L2**	0.681	0.316	0.818	0.224	0.839		0.851	
**L3**	0.716	0.359	0.764	0.195	0.815		0.809	
**L4**	0.422	0.581	0.719	0.358	0.785		0.78	
**L6**	0.242	0.607	0.624	0.446	0.733		0.733	
**L7**	0.399	0.614	0.853	0.197	0.826		0.861	
**L8**	0.430	0.575	0.61	0.216	0.840		0.671	
**L5**	-0.141	-0.296	0.17	0.685		1.097		0.661
**L9**	-0.029	-0.187	0.193	0.655		0.267		0.682

Note that a standardized factor loading above 1 may indicate multicollinearity between factors (http://www.ssicentral.com/lisrel/techdocs/HowLargeCanaStandardizedCoefficientbe.pdf).

In general, items L1-L4 and L6-L8 were highly, positively correlated, with items L5, L9, and L10 having negative or null associations with other items.

S2 Table. Spearman's correlation coefficient between Vaccine Hesitancy Scale Items

	**L1**	**L2**	**L3**	**L4**	**L6**	**L7**	**L8**	**L5**	**L9**	**L10**
**L1**		0.61**	0.59**	0.49**	0.38**	0.51**	0.52**	-0.10	-0.13**	0.11
**L2**			0.71**	0.60**	0.52**	0.59**	0.56**	-0.19**	-0.09	0.09
**L3**				0.62**	0.50**	0.59**	0.63**	-0.21**	-0.12**	0.11
**L4**					0.57**	0.57**	0.55**	-0.26**	-0.13**	0.03
**L6**						0.59**	0.58**	-0.33**	-0.14**	0.03
**L7**							0.72**	-0.22**	-0.11	0.12**
**L8**								-0.22**	-0.17**	0.08
**L5**									0.25**	0.17**
**L9**										0.21**

** P<0.0001 *P<0.05
